# Ommaya Reservoir-Based Cerebrospinal Fluid Chemotherapy for Leptomeningeal Metastasis in the Era of Targeted Therapy: Safety, Survival, and Exploratory Prognostic Factors from a Single-Center Cohort

**DOI:** 10.3390/jcm15207808

**Published:** 2026-10-10

**Authors:** Huaixu Li, Hongqing Cai, Xiaohong Lu, Qi Liu, Yujia Chen, Yulin Mi, Jinghai Wan

**Affiliations:** 1 Department of Neurosurgery, National Cancer Center/National Clinical Research Center for Cancer/Cancer Hospital, Chinese Academy of Medical Sciences and Peking Union Medical College, Beijing 100021, China; lihuaixu1997@163.com (H.L.); pumc67@outlook.com (Q.L.); camuelchen@126.com (Y.C.); 2 Department of Medical Oncology, Sanhuan Cancer Hospital, Chaoyang District, Beijing 100021, China; lxhaa2020@163.com

**Keywords:** leptomeningeal metastasis, Ommaya reservoir, cerebrospinal fluid-directed chemotherapy, prognosis, performance status

## Abstract

**Objective:** Real-world data on Ommaya reservoir-based cerebrospinal fluid (CSF) chemotherapy for leptomeningeal metastasis (LM) in the targeted-therapy era are limited. We describe its safety and survival and explore prognostic factors in lung adenocarcinoma-associated LM. **Methods:** We retrospectively reviewed 68 patients with LM treated with Ommaya reservoir implantation and CSF chemotherapy. Overall survival (OS) was estimated from LM diagnosis and from implantation, and an exploratory multivariable Cox regression was performed in patients with actionable driver mutations receiving targeted therapy. **Results:** Device-related complications occurred in six patients (8.8%), including three catheter malpositions after freehand placement; chemotherapy-related adverse effects occurred in four (5.9%). After a median follow-up of 12.8 months, 14 patients (20.6%) had died, 10 of CNS progression. Median OS was not reached from diagnosis (24-month OS 67.8%) and 18.7 months from implantation (24-month OS 48.3%). In the exploratory analysis (*n* = 46), KPS, age, and EGFR mutation subtype were associated with OS from both time origins. **Conclusions:** Ommaya reservoir-based CSF chemotherapy was feasible with few device-related complications. Because all patients were treated and most had lung adenocarcinoma receiving targeted therapy, the favorable survival cannot be attributed to the reservoir or generalized to other primary tumors; prognostic factors require prospective validation.

## 1. Introduction

Leptomeningeal metastasis (LM) is a severe central nervous system complication of advanced solid tumors, characterized by the dissemination of malignant cells into the subarachnoid space and pia mater, leading to multifocal neurological deficits and a marked decline in quality of life [1]. Previous studies and clinical practice guidelines indicate that, although LM is considered “treatable but generally incurable,” the median overall survival typically remains only a few months even with multimodal therapy; nonetheless, active treatment is still recommended after careful clinical assessment in order to prolong survival as much as possible and alleviate symptoms [2,3].

Currently, treatment strategies for LM mainly include systemic therapy and cerebrospinal fluid-directed chemotherapy. Systemic therapy comprises targeted therapy, immunotherapy, and conventional chemotherapy, and must balance control of both intracranial and extracranial disease with adequate penetration of the blood–brain barrier [3,4]. Cerebrospinal fluid (CSF) chemotherapy is an important local treatment modality; guideline-recommended intrathecal agents commonly include methotrexate, cytarabine and its sustained-release formulations, and thiotepa. In recent years, newer agents such as pemetrexed have also been administered intrathecally or intraventricularly for LM from solid tumors, and have shown promising intracranial response rates with acceptable toxicity in patients with EGFR-mutated non-small cell lung cancer-associated LM [5,6,7].

CSF-directed chemotherapy is mainly delivered via lumbar puncture or intraventricular administration through an Ommaya reservoir. Multiple guidelines and clinical studies suggest that drug delivery via an Ommaya reservoir provides a more homogeneous distribution of chemotherapeutic agents within the CSF, more stable exposure profiles, and better patient adherence, and is therefore recommended as the preferred route [1,8,9]. However, Ommaya reservoir implantation is an invasive neurosurgical procedure that may lead to complications such as infection, hemorrhage, hydrocephalus, and device malfunction. These risks continue to raise safety concerns among clinicians, resulting in a discrepancy between guideline recommendations and limited use in routine practice [10,11]. In the era of modern targeted and immune therapies, real-world data on the survival outcomes and safety of Ommaya reservoir-based CSF chemotherapy, and on the characteristics of the patients treated with this approach, remain scarce.

Based on a recent cohort of patients with LM treated at our center, this study aimed to evaluate overall survival (OS) outcomes and the safety profile of Ommaya reservoir-mediated CSF chemotherapy. Furthermore, we sought to explore clinical and molecular factors associated with survival among treated patients with lung adenocarcinoma-associated LM, as a hypothesis-generating step toward future prospective studies of patient selection.

## 2. Materials and Methods

### 2.1. Study Design

This retrospective single-center cohort study included 68 patients with leptomeningeal metastasis who underwent Ommaya reservoir implantation at the Cancer Hospital, Chinese Academy of Medical Sciences between January 2024 and July 2025. All patients received at least one intrathecal treatment and had complete clinical and follow-up data. Patients younger than 18 years, those without definitive LM diagnosis, or with missing key clinical data were excluded. The study was approved by the institutional ethics committee, and patient data were anonymized prior to analysis.

### 2.2. Diagnosis of LM and Data Collection

The diagnosis of LM was established based on CSF cytology reporting positive malignant cells [12]. Clinical data were obtained from electronic medical records and supplemented by outpatient or telephone follow-up. Collected variables included demographic characteristics, primary tumor type, history of primary tumor resection, extracranial and intracranial metastases, EGFR mutation status in lung adenocarcinoma, and use of targeted therapy and intrathecal treatment. LM-related symptoms at diagnosis were recorded. All patients underwent Ommaya reservoir placement, with preoperative Karnofsky Performance Status (KPS) and Neurologic Assessment in Neuro-Oncology (NANO) scores documented, as well as postoperative complications. Treatment variables included intrathecal agents and number of administrations. Hydrocephalus on neuroimaging and follow-up data on symptom evolution and survival were also collected. Opening pressure at the diagnostic lumbar puncture was not routinely measured in this cohort; clinical or radiological features suggestive of raised intracranial pressure (ICP) during intrathecal treatment, and their management, were documented. Detailed treatment information was recorded, including intrathecal agents and dose, systemic therapy (targeted agents and their generation, chemotherapy, immunotherapy, and antiangiogenic therapy), whole-brain radiotherapy, molecular subtype, the interval from LM diagnosis to reservoir implantation, and whether LM was diagnosed during targeted therapy. For deceased patients, the cause of death was classified from medical records and telephone follow-up as CNS progression (leptomeningeal or parenchymal progression with neurological deterioration), systemic progression, or treatment-related.

### 2.3. Treatment Procedures

Ommaya reservoir implantation was performed by an experienced neurosurgical team following a standardized surgical approach. A frontal ventricular catheter was placed through a burr hole at Kocher’s point and connected to a subgaleal reservoir. Frameless neuronavigation was used in 38 patients (55.9%) and freehand placement based on anatomical landmarks in 30 (44.1%); because a single navigation system is shared among operations performed concurrently, the choice was made at the discretion of the operating surgeon rather than by protocol. Catheter position was confirmed by computed tomography (CT) on the first postoperative day in all patients. Before each intrathecal administration, a volume of CSF equal to the injection volume was withdrawn slowly under strict aseptic technique, and the drug was instilled slowly to avoid abrupt fluctuations in ICP. Intrathecal methotrexate was given at 10 mg per dose, pemetrexed at 30 mg per dose with folic acid and vitamin B12 supplementation, and thiotepa at 10 mg per dose; the frequency of administration was individualized according to clinical and cytological response. Postoperative intrathecal regimens were individualized based on tumor type and molecular characteristics. Systemic treatments including targeted therapy, immunotherapy, and chemotherapy were prescribed by the attending oncologist according to prior therapies and overall patient condition.

### 2.4. Endpoints and Follow-Up

The primary endpoint was OS, defined as the time from the diagnosis of leptomeningeal metastasis to death from any cause or the last follow-up. Patients who remained alive at the last follow-up were censored on that date. As a secondary endpoint, OS was also calculated from the date of Ommaya reservoir implantation to account for the interval between diagnosis and implantation. Complications were classified as device/procedure-related (attributable to implantation or to the reservoir itself, including catheter malposition, hemorrhage, subdural effusion, wound or device-associated infection, and device malfunction) or as adverse effects of intrathecal chemotherapy (attributable to the drug, including chemical meningitis, myelosuppression, and neurotoxicity such as leukoencephalopathy).

### 2.5. Statistical Analysis

Baseline characteristics were summarized using descriptive statistics. Continuous variables are presented as median and range, and categorical variables are presented as number and percentage. OS was estimated using the Kaplan–Meier method and compared using the log-rank test. Associations between clinical variables and OS were assessed using Cox proportional hazards regression models.

For the exploratory multivariable analysis, analyses were restricted to patients with lung adenocarcinoma-associated LM harboring actionable driver mutations who had received matched targeted therapy. Because only 10 deaths occurred in this subgroup, the multivariable analysis was regarded as exploratory and hypothesis-generating. Variables were entered into multivariable Cox regression on the basis of univariable results (age and KPS) or pre-specified clinical relevance (epilepsy, hydrocephalus, extracranial metastasis, and EGFR mutation subtype), and the four variables retained (age, KPS, extracranial metastasis, and EGFR mutation subtype) formed the model used for the nomogram; this model was also fitted with OS measured from implantation as a sensitivity analysis. Discrimination was summarized by Harrell’s C-index with optimism correction by bootstrap resampling (1000 resamples); time-dependent ROC analysis was not used because it is unreliable with few events. The nomogram and a risk-score stratification (tertiles of the linear predictor) are shown for illustration only in the Supplementary Material and are not intended for clinical use before external validation. All tests were two-sided, with *p* < 0.05 considered statistically significant. Statistical analyses were performed using R version 4.4.1 (R Foundation for Statistical Computing, Vienna, Austria) with the survival (version 3.8.3), survminer (version 0.4.9), and rms (version 6.8.2) packages.

## 3. Results

### 3.1. Baseline Characteristics

A total of 68 patients with leptomeningeal metastasis treated with Ommaya reservoir implantation were included. The median age was 61 years (range, 37–79), and 41 patients (60.3%) were female. Median pre-procedural KPS and NANO scores were 90 (range, 10–100) and 2 (range, 0–8), respectively. Lung adenocarcinoma was the predominant primary tumor (85.3%). Most patients had additional metastatic disease at LM diagnosis, including brain parenchymal metastases in 69.1% and extracranial metastases in 54.4%. Headache and focal neurological deficits were the most common presenting symptoms. Hydrocephalus was identified in 14.7% of patients. Targeted therapy was administered in 76.5% of cases, and intrathecal chemotherapy mainly consisted of methotrexate or pemetrexed. Baseline characteristics are summarized in Table 1. Treatment details are summarized in Table 2. Among the 58 patients with lung adenocarcinoma, 16 harbored EGFR exon 19 deletions, 20 L858R, three uncommon EGFR mutations, and three EGFR mutations of unspecified subtype; two had ALK rearrangements, three ERBB2 alterations, one a KRAS mutation, and 10 had no actionable driver. Thirty-seven patients (63.8%) received a third-generation EGFR-TKI, and LM was diagnosed during TKI therapy in 46 of the 48 TKI-treated patients. Whole-brain radiotherapy had been given in six patients (8.8%). The median interval from LM diagnosis to reservoir implantation was 33 days (interquartile range, 16–84; range, 2–1214), and 15 patients (22.1%) were implanted more than 90 days after diagnosis, mostly after initial systemic therapy at other institutions.

### 3.2. Device-Related Complications and Intracranial Pressure

All 68 patients successfully underwent Ommaya reservoir placement and received CSF chemotherapy. Device-related complications occurred in six patients (8.8%): catheter malposition requiring revision in three (4.4%), subdural effusion in two (2.9%), and CSF infection during intrathecal therapy in one (1.5%). All three malpositions occurred among the 30 freehand placements (10.0%) and none among the 38 navigation-guided placements (0%; Fisher’s exact test, *p* = 0.08); they were identified on the routine CT on the first postoperative day in patients with non-dilated ventricles and were revised in the operating room, and in one patient the device was replaced. No malposition occurred among the 10 patients with hydrocephalus. The subdural effusions resolved with conservative management, and the CSF infection, which occurred 2 months after implantation, was treated with antimicrobial therapy without device removal. No hemorrhage, wound complication, device malfunction, or perioperative death occurred.

Opening pressure was measured at the diagnostic lumbar puncture in only four patients (89–330 mm H_2_O; >200 mm H_2_O in one) and was not measured at implantation. Radiological hydrocephalus was present in 10 patients (14.7%), one of whom required ventriculoperitoneal shunting, and three patients (4.4%) developed clinical or radiological features of raised ICP during intrathecal treatment, managed with osmotherapy (*n* = 2) or ventriculoperitoneal shunting (*n* = 1).

### 3.3. Adverse Effects of Intrathecal Chemotherapy

Adverse effects attributable to intrathecal chemotherapy rather than to the device were recorded in four patients (5.9%): grade 3 myelosuppression (*n* = 1), thrombocytopenia (*n* = 1), vomiting (*n* = 1), and one probable methotrexate-related pseudotumoral demyelinating lesion (described below). All resolved after supportive treatment or discontinuation of the agent. These events are reported separately from device-related complications because they reflect drug toxicity rather than the reservoir itself.

#### Representative Case: A Probable Pseudotumoral Demyelinating Lesion

A 60-year-old woman with ER/PR-positive, HER2-negative breast cancer developed leptomeningeal metastasis after prior treatment for bone metastases. She had previously undergone whole-brain radiotherapy. At the time of LM diagnosis, brain MRI demonstrated diffuse leptomeningeal enhancement, CSF cytology was positive, the KPS score was 80, and muscle strength of all extremities was grade V. The patient was then treated with intrathecal methotrexate administered via an Ommaya reservoir in combination with systemic chemotherapy, resulting in initial neurological improvement. However, during prolonged intrathecal methotrexate therapy via the Ommaya reservoir, she gradually developed neurological deterioration. Follow-up brain MRI revealed newly developed mass-like white matter lesions, while PET-CT showed hypometabolism (Figure 1). No evidence of disease progression was observed in peripheral blood or CSF, and the lesions were therefore considered unlikely to represent tumor progression. Overall, the findings were consistent with a probable pseudotumoral demyelinating lesion, regarded as probable methotrexate-related neurotoxicity (leukoencephalopathy) after prolonged intrathecal administration, rather than as a complication of the reservoir itself. Intrathecal methotrexate was discontinued, after which both neurological symptoms and imaging findings remained stable without further progression. At the last follow-up, the patient was alive 15 months after the diagnosis of LM.

### 3.4. Overall Survival in the Entire Cohort

By the data cutoff, the median follow-up duration for the entire cohort was 12.8 months (range, 1.3 to 53.9 months). A total of 14 deaths (20.6%) were documented. The cause of death was CNS progression in 10 patients (71.4%) and systemic progression in four (28.6%) (Supplementary Table S2); no death was attributed to the reservoir or to intrathecal chemotherapy. The Kaplan–Meier survival curve showed that the overall survival probability remained above 50% throughout follow-up, and the median overall survival had not been reached (Figure 2A). Kaplan–Meier-estimated time-specific overall survival rates were approximately 86.4%, 84.4%, and 67.8% at 6, 12, and 24 months after LM diagnosis, respectively. When survival was measured from reservoir implantation, the median follow-up was 9.9 months (range, 0.3 to 23.9), the median OS was 18.7 months (95% CI, 18.7 to not reached), an estimate driven by a single late event with only three patients still at risk, and the 6-, 12-, and 24-month OS rates were 84.7%, 82.6%, and 48.3%, respectively (Supplementary Figure S1A). OS did not differ significantly among patients with lung adenocarcinoma harboring actionable driver mutations who received targeted therapy (*n* = 46; 10 deaths; median OS not reached), patients with lung adenocarcinoma without targeted therapy (*n* = 12; 3 deaths; median OS 19.5 months), and patients with other primary tumors (*n* = 10; 1 death; median OS not reached) (log-rank *p* = 0.6 from diagnosis and *p* = 0.8 from implantation; Supplementary Figure S1B,C), although the number of events in the latter two groups was too small for a meaningful comparison.

### 3.5. Exploratory Dichotomized Survival Analyses in Lung Adenocarcinoma with LM

To characterize survival heterogeneity in patients with lung adenocarcinoma-associated leptomeningeal metastasis, Kaplan–Meier analyses were performed in all 58 patients. In an exploratory analysis using an age cutoff of 50 years, patients aged < 50 years had significantly shorter overall survival compared with those aged ≥ 50 years (Figure 2B). Sex was not significantly associated with overall survival, although a trend toward poorer survival was observed in male patients (Figure 2C). Neither epilepsy nor hydrocephalus was significantly associated with overall survival (Figure 2D,E). Performance status showed a strong association with survival. Patients with a KPS ≥70 had significantly longer overall survival than those with KPS < 70 (Figure 2F). A lower NANO score (<5) showed a trend toward improved survival compared with a NANO score ≥ 5 (Figure 2G). Among treatment-related factors, receipt of ≥6 cycles of intrathecal (CSF) chemotherapy was associated with a nonsignificant trend toward longer overall survival compared with fewer cycles (Figure 2H). When survival was measured from reservoir implantation, the associations with age (log-rank *p* = 0.032) and KPS (*p* < 0.001) persisted, the NANO score reached significance (*p* = 0.043), and sex, epilepsy, hydrocephalus, and the number of intrathecal injections remained non-significant (Supplementary Table S3; Supplementary Figure S2).

### 3.6. Exploratory Univariable and Multivariable Analyses in Patients with Lung Adenocarcinoma-Associated LM

Given that patients with driver gene-positive lung adenocarcinoma receiving targeted therapy constituted the largest proportion of the LM cohort, the study population was further restricted to this specific subgroup in the univariable and multivariable Cox regression analyses. This approach was adopted to reduce confounding arising from treatment heterogeneity. Because only 10 deaths occurred in this subgroup, the multivariable analysis was regarded as exploratory and hypothesis-generating. Only patients harboring actionable driver mutations who had previously received the corresponding targeted therapy were included (*n* = 46). Actionable driver mutations included EGFR, ALK, and other targetable alterations.

The results of the univariable Cox regression analyses are summarized in Table 3. Age and KPS entered the multivariable model on the basis of these results, and epilepsy, hydrocephalus, extracranial metastasis, and mutation subtype on the basis of pre-specified clinical relevance (Table 4).

For the multivariable analysis, mutation type was dichotomized according to common clinical practice: classical EGFR mutations (exon 19 deletion or exon 21 L858R) were classified as one group, and all other mutation types as the reference group. In the exploratory multivariable Cox regression, higher KPS was associated with a lower risk of death (*p* = 0.0003), whereas the presence of extracranial metastases was associated with a significantly increased risk of death (*p* = 0.044). Compared with other mutation types, patients with classical EGFR mutations had significantly better survival outcomes (*p* = 0.014). Age was also significantly associated with overall survival (*p* = 0.008). In the four-variable model used for the nomogram, age (HR 0.89, 95% CI 0.82–0.96), KPS (HR 0.91, 95% CI 0.86–0.96), and classical EGFR mutation (HR 0.09, 95% CI 0.01–0.64) remained associated with OS, whereas extracranial metastasis did not reach significance (HR 9.3, 95% CI 0.96–89.9; *p* = 0.054). When OS was measured from implantation, KPS (HR 0.91, 95% CI 0.87–0.96), age (HR 0.91, 95% CI 0.84–0.98), and mutation subtype (HR 0.18, 95% CI 0.03–0.98) remained associated with OS, but extracranial metastasis did not (HR 3.0, 95% CI 0.38–23.6; *p* = 0.30) (Supplementary Table S4). The confidence intervals were wide (10 events among 46 patients; 2.5 events per variable).

### 3.7. Exploratory Prognostic Model

For illustration only, the four-variable model (age, KPS, extracranial metastasis, and mutation subtype) was presented as a nomogram for 1-, 2-, and 3-year overall survival (Supplementary Figure S3A). The apparent Harrell’s C-index was 0.945 (standard error 0.020), and the bootstrap optimism-corrected C-index was 0.909 (0.913 when OS was measured from implantation).

Risk tertiles derived from the linear predictor contained zero, one, and nine deaths, respectively (log-rank *p* < 0.001; Supplementary Figure S3B).

### 3.8. Survival Outcomes in Patients with Breast Cancer-Associated LM

Five patients with breast cancer-associated LM were included. During follow-up, only one patient died, and the median overall survival was not reached. Two patients developed device-related complications and one an adverse effect of intrathecal methotrexate during treatment; all were managed symptomatically and were able to complete CSF chemotherapy, with no evident long-term functional impairment observed. Clinical details of patients with breast cancer-associated LM are summarized in Supplementary Table S1.

## 4. Discussion

Ommaya reservoir-mediated CSF-directed chemotherapy has been widely recommended by major guidelines for selected patients with LM, largely because it provides reliable ventricular access, enables standardized and sustained intrathecal drug delivery, and facilitates coordination with systemic therapy [1,2]. In this single-center retrospective study conducted in the era of current systemic treatments, we systematically evaluated survival outcomes and safety profiles of LM patients treated with Ommaya reservoir implantation and CSF chemotherapy. Our data describe the safety profile and survival of a contemporary, selected cohort treated with this approach; because all patients received reservoir-based therapy, they do not allow inferences about the survival benefit of the reservoir itself or about which patients benefit from it.

A common concern in routine practice is the potential morbidity associated with reservoir implantation and its subsequent use [1]. In our cohort, device-related complications occurred in six patients (8.8%), none of which required permanent device removal or caused permanent deficit, and there were no perioperative deaths. Catheter malposition (3/68, 4.4%) was the most frequent device-related event. This rate is comparable to that reported in earlier series of Ommaya reservoirs for LM [13], but it is higher than what can be achieved with image guidance: freehand ventricular catheter placement misses the ventricle in more than 20% of attempts [14], particularly when the ventricles are not dilated, as was the case in all three of our patients and as is typical in LM, whereas image-guided placement substantially improves accuracy. In our cohort, neuronavigation was used at the surgeon’s discretion, because a single system is shared among concurrent operations, and all three malpositions occurred among the 30 freehand placements, with none among the 38 navigation-guided placements (10% versus 0%); with so few events this difference did not reach statistical significance, but it is consistent with the published data. Although the revisions were uneventful, each revision is an additional procedure with its own risk. We therefore consider image guidance the standard of care for Ommaya placement in patients with normal-sized or small ventricles.

The remaining device-related events, CSF infection and subdural effusion, likely reflect, at least in part, modifiable factors related to reservoir handling and CSF dynamics during intrathecal administration. Consistent with prior reports, Ommaya reservoir-associated infections can often be successfully managed with appropriate antimicrobial therapy without routine reservoir removal, thereby allowing continuation of intrathecal and systemic anticancer treatment in selected patients [15,16]. These findings emphasize two key technical principles in daily practice, namely strict aseptic technique during every access to minimize infectious risk, as well as slow and controlled CSF withdrawal and drug instillation to avoid abrupt intracranial pressure fluctuations that may predispose patients to complications such as subdural collections. In this context, the primary goal of complication management is to preserve treatment continuity while ensuring procedural safety. Intracranial pressure also deserves attention: uncontrolled ICP was an unfavorable prognostic factor in a large series of patients receiving intraventricular chemotherapy for lung cancer-associated LM [17]. The reservoir itself provides a route for gentle CSF withdrawal before drug instillation and for pressure monitoring, and persistent elevation should prompt evaluation for CSF diversion before intrathecal therapy is continued. Because opening pressure was not measured systematically in our cohort, we now recommend its routine measurement at the diagnostic lumbar puncture.

Importantly, we identified one case of a pseudotumoral demyelinating lesion during prolonged intrathecal methotrexate therapy. This event is best regarded as methotrexate-related neurotoxicity (leukoencephalopathy) rather than as a complication of the reservoir, and it is therefore reported separately from device-related complications; methotrexate-induced leukoencephalopathy is a well-characterized entity whose MRI changes may persist after treatment [18]. Radiologically, this lesion closely mimicked tumor progression and could be easily misdiagnosed, which might result in inappropriate treatment escalation or premature changes in systemic or intrathecal therapy when imaging findings are considered in isolation. Comprehensive evaluation including metabolic imaging and the absence of concordant progression in systemic assessment and CSF studies supported a non-neoplastic etiology. This case highlights the need for careful differential diagnosis when new enhancing parenchymal lesions emerge in patients receiving Ommaya-based intrathecal therapy, and underscores that treatment assessment should integrate clinical evolution, imaging patterns, and multimodal evaluation rather than relying on morphology alone. When such neurotoxicity is suspected, methotrexate should be discontinued and, where appropriate, replaced by an alternative agent such as pemetrexed.

From the perspective of survival outcomes, LM has historically been associated with dismal outcomes, with prior real-world studies and guideline-based estimates typically reporting median overall survival of approximately 3–6 months [19]. In contrast, the median overall survival from LM diagnosis had not been reached in our cohort, with a 24-month survival rate approaching 70%. This favorable outcome must be interpreted with caution and should not be attributed to reservoir-based therapy. First, 85% of patients had lung adenocarcinoma and 76% received targeted therapy, mostly CNS-penetrant third-generation EGFR-TKIs; although OS did not differ significantly between this subgroup and the remaining 22 patients, the latter experienced only four deaths, so the study cannot address the contribution of systemic therapy. Second, patients were selected for surgery and had a median KPS of 90 at implantation. Third, OS measured from LM diagnosis includes the interval before implantation, during which patients had to survive to enter the cohort; 22% of patients were implanted more than 90 days after diagnosis, and when OS was measured from implantation the median OS was 18.7 months and the 24-month OS fell from 67.8% to 48.3%. Fourth, follow-up was short (median 9.9 months from implantation), and survival estimates beyond 18 months rest on very few patients at risk. Finally, 10 of the 14 deaths were attributed to CNS progression and four to systemic progression, indicating that, despite intrathecal therapy, control of CNS disease remained the main determinant of mortality in this cohort. Without a comparison group, the contribution of intrathecal therapy cannot be separated from that of systemic therapy, and these results should not be generalized to LM from breast cancer, other solid tumors, or driver-negative lung cancer.

From a clinical perspective, understanding which factors are associated with survival after Ommaya reservoir implantation is relevant to counseling and treatment planning. Regarding prognostic determinants, performance status emerged as the most consistent predictor of overall survival. KPS remained independently associated with survival in the univariable and multivariable analyses and in the exploratory model, supporting its central role in prognostic assessment [20,21]. In the subgroup of lung adenocarcinoma patients harboring actionable driver mutations and receiving corresponding targeted therapy, age, extracranial metastases, and mutation type further contributed to risk stratification, consistent with the prior literature emphasizing the impact of systemic disease burden and molecular background on LM prognosis [22,23,24]. In our cohort, patients with a constellation of unfavorable prognostic features, including younger age, poor performance status, non-classical EGFR mutations, and the presence of extracranial metastases, were more frequently classified into higher-risk groups by the exploratory model and had poorer survival despite intensive CSF-directed therapy. The apparent separation of these risk groups is expected when a model is evaluated in the data used to build it and does not indicate predictive validity. These associations were derived from an exploratory analysis of 46 patients with only 10 deaths, and the wide confidence intervals reflect this limitation; notably, the association with extracranial metastasis was not robust to the choice of time origin, whereas performance status, age, and EGFR mutation subtype were. In practice, these factors can inform counseling about expected survival after reservoir implantation and may help stratify patients in future prospective studies. Because all patients in this cohort were treated, however, they cannot by themselves identify who benefits from the reservoir; this question requires studies with an appropriate comparison group.

Nevertheless, this study has several limitations. First, and most importantly, all patients received reservoir-based intrathecal therapy and there was no comparison group, so the benefit of the reservoir, and which patients benefit from it, cannot be inferred. Second, the retrospective single-center design and the selection of patients for surgery introduce selection bias, and measuring OS from LM diagnosis introduces immortal time, which is why survival from implantation is also reported. Third, the cohort was dominated by lung adenocarcinoma with actionable driver mutations treated with targeted therapy, which limits generalizability to LM from other primary tumors and probably contributes to the favorable survival. Fourth, the number of deaths was small, so the multivariable analysis is at risk of overfitting and should be regarded as hypothesis-generating; it was not pre-specified and has not been externally validated. Finally, treatments were individualized, the number of intrathecal injections is a time-dependent variable subject to immortal-time bias, ICP was not systematically measured, image guidance was used at the surgeon’s discretion, the cause of death was classified from clinical records rather than by autopsy, and follow-up was short, so the heterogeneity of treatment could not be fully adjusted for and survival estimates remain immature.

## 5. Conclusions

In this single-center retrospective cohort, Ommaya reservoir-based CSF chemotherapy was feasible, with a low rate of device-related complications. Survival was longer than historically reported for LM, but this should be viewed in the context of a cohort dominated by lung adenocarcinoma with actionable driver mutations treated with targeted therapy and of short follow-up. Because all patients received reservoir-based therapy, these findings describe the outcomes of treated patients and do not establish the benefit of the reservoir itself, which requires controlled studies. Performance status, age, and EGFR mutation subtype were associated with survival from both LM diagnosis and reservoir implantation in an exploratory analysis and warrant validation in larger, prospective, multicenter cohorts.

## Figures and Tables

**Figure 1:**
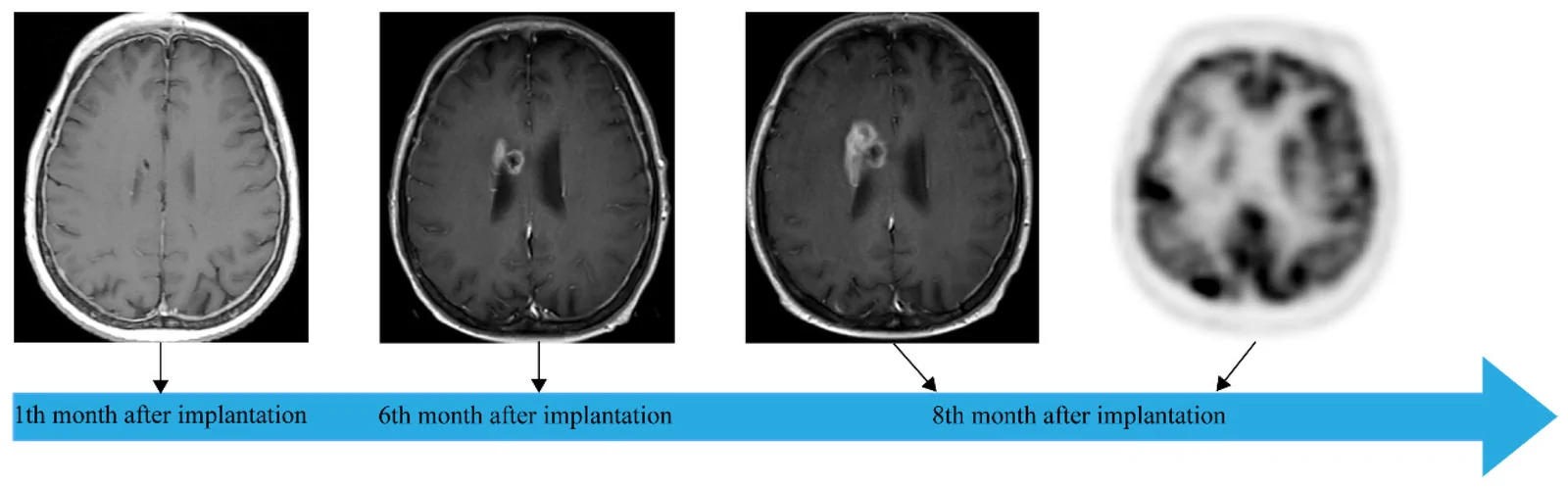
Serial brain MRI and PET-CT of a patient with leptomeningeal metastasis who developed a pseudotumoral demyelinating lesion, consistent with probable methotrexate-related neurotoxicity, during prolonged intrathecal methotrexate therapy via an Ommaya reservoir. Contrast-enhanced T1-weighted MRI showed no focal parenchymal abnormality at 1 month after implantation, followed by the development of a mass-like white matter lesion with ring enhancement at 6 months. At 8 months after implantation, the lesion persisted without further progression after discontinuation of intrathecal methotrexate. Corresponding FDG PET-CT at the same time point demonstrated hypometabolism of the lesion, supporting a non-neoplastic etiology. Abbreviations: MRI, magnetic resonance imaging; PET-CT, positron emission tomography/computed tomography; FDG, fluorodeoxyglucose.

**Figure 2:**
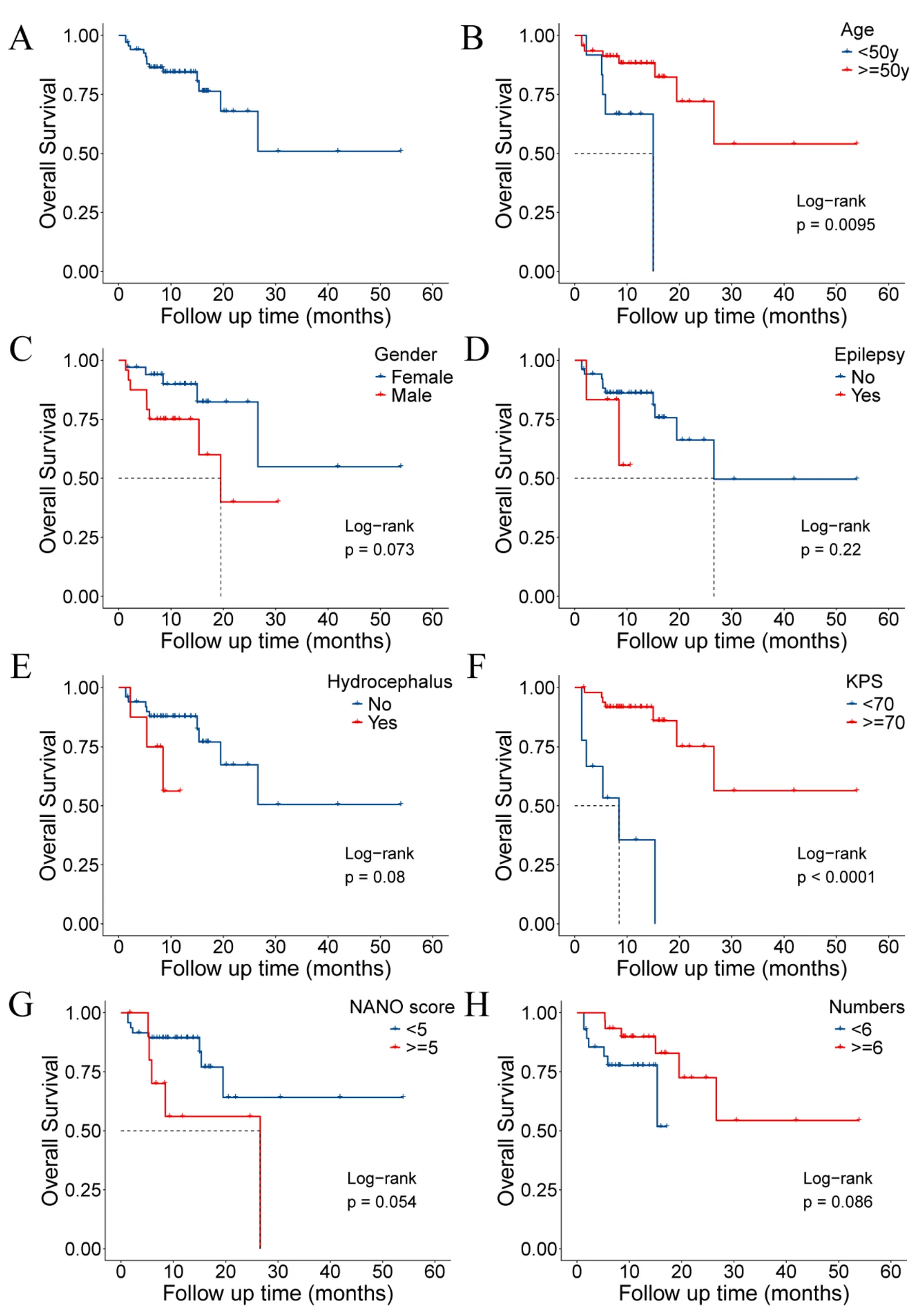
Kaplan–Meier survival analyses (overall survival from LM diagnosis). (**A**). Overall survival of the entire cohort (*n* = 68). (**B**–**H**). Patients with lung adenocarcinoma-associated leptomeningeal metastasis (*n* = 58). (**B**). Overall survival stratified by age (<50 vs. ≥50 years). (**C**). Overall survival stratified by gender. (**D**). Overall survival stratified by the presence or absence of epilepsy at the time of LM diagnosis. (**E**). Overall survival stratified by the presence or absence of hydrocephalus on neuroimaging at LM diagnosis. (**F**). Overall survival stratified by baseline KPS (KPS <70 vs. ≥70). (**G**). Overall survival stratified by baseline NANO score (≥5 vs. <5). (**H**). Overall survival stratified by the number of CSF chemotherapy injections received (<6 vs. ≥6). *p* values were calculated using the log-rank test. Dashed lines indicate the median survival time.

**Table 1:** Baseline characteristics of 68 patients with leptomeningeal metastasis treated with Ommaya reservoir.

Characteristics		*N* (%)
Age	Median	61
Gender	Male	27 (39.7)
	Female	41 (60.3)
KPS	Median	90
NANO Score	Median	2 (0–8)
Primary Tumor	Lung adenocarcinoma	58 (85.3)
	Pleomorphic carcinoma	1 (1.5)
	Atypical carcinoid	1 (1.5)
	Small-cell lung cancer	1 (1.5)
	Glioblastoma	1 (1.5)
	Ovarian cancer	1 (1.5)
	Breast cancer	5 (7.4)
Surgery for primary tumor	Yes	29 (42.6)
	No	39 (57.4)
Metastasis	Any metastasis other than LM	57 (83.8)
	Brain metastasis	47 (69.1)
	Extracranial metastasis	37 (54.4)
Symptoms	Headache	34 (50.0)
	Epilepsy	6 (8.8)
	Focal symptoms	43 (63.2)
Device-related complications	Catheter malposition requiring revision	3 (4.4)
Subdural effusion		2 (2.9)
	CSF infection	1 (1.5)
Intrathecal chemotherapy-related adverse events	Any (see Section 3.3)	4 (5.9)
Hydrocephalus	Yes	10 (14.7)
	No	58 (85.3)
Targeted therapy	Yes	52 (76.5)
	No	16 (23.5)
CSF chemotherapy drugs	MTX	24 (35.3)
	PEM	19 (27.9)
	PEM following MTX resistance	20 (29.4)
	Thiotepa	5 (7.4)

Values are presented as median (range) or number (%), unless otherwise specified. Abbreviations: KPS, Karnofsky Performance Status; NANO, Neurologic Assessment in Neuro-Oncology; LM, leptomeningeal metastasis; CSF, cerebrospinal fluid; MTX, methotrexate; PEM, pemetrexed.

**Table 2:** Treatment details of 68 patients with leptomeningeal metastasis treated with Ommaya reservoir.

Characteristics		*N* (%) or Median (Range)
Reservoir placement (*n* = 68)		
	Navigation-guided	38 (55.9)
	Freehand (anatomical landmarks)	30 (44.1)
	Catheter malposition requiring revision: freehand/navigation-guided	3 (10.0)/0 (0)
Intrathecal regimen (*n* = 68)		
	Methotrexate, dose per administration (mg)	10
	Pemetrexed, dose per administration (mg)	30
	Thiotepa, dose per administration (mg)	10
	Number of intrathecal administrations	4.5 (1–39)
	≥6 intrathecal administrations	33 (48.5)
Systemic therapy for LM (*n* = 68)		
	Targeted therapy	52 (76.5)
	Chemotherapy	55 (80.9)
	Immunotherapy	29 (42.6)
	Antiangiogenic therapy	44 (64.7)
	No systemic therapy	2 (2.9)
Targeted agents, lung adenocarcinoma (*n* = 58)		
	Third-generation EGFR-TKI (osimertinib, furmonertinib, aumolertinib)	37 (63.8)
	First- or second-generation EGFR-TKI only	6 (10.3)
	ALK inhibitor	2 (3.4)
	Other targeted agent	2 (3.4)
	Targeted agent not specified	1 (1.7)
	No targeted therapy	10 (17.2)
	LM diagnosed during TKI therapy (of 48 TKI-treated)	46 (95.8)
Radiotherapy (*n* = 68)		
	Whole-brain radiotherapy	6 (8.8)
Molecular subtype, lung adenocarcinoma (*n* = 58)		
	EGFR exon 19 deletion	16 (27.6)
	EGFR L858R	20 (34.5)
	Uncommon EGFR mutation (G719A, exon 20 insertion)	3 (5.2)
	EGFR mutation, subtype not specified	3 (5.2)
	ALK rearrangement	2 (3.4)
	ERBB2 alteration	3 (5.2)
	KRAS mutation	1 (1.7)
	No actionable driver/other	10 (17.2)
Treatment timing (*n* = 68)		
	LM diagnosis to reservoir implantation, days	33 (2–1214)
	Implantation > 90 days after LM diagnosis	15 (22.1)

Values are presented as number (%) or median (range). Focal radiotherapy and the schedule of intrathecal administration were not systematically recorded. Abbreviations: LM, leptomeningeal metastasis; EGFR, epidermal growth factor receptor; TKI, tyrosine kinase inhibitor; ALK, anaplastic lymphoma kinase.

**Table 3:** Univariable Cox proportional hazards analysis of overall survival.

Variable	HR	95% CI	*p*-Value
Gender	1.86	0.53–6.54	0.336
Age	0.94	0.88–0.99	0.028 *
Headache	1.29	0.37–4.55	0.693
Epilepsy	2.18	0.45–10.58	0.332
Hydrocephalus	2.86	0.55–14.80	0.209
KPS	0.96	0.94–0.98	0.0002 *
Brain metastasis	3.64	0.45–29.26	0.224
Extracranial metastasis	2.20	0.57–8.55	0.255
NANO	1.38	0.98–1.94	0.063
Surgery	1.15	0.32–4.11	0.828
Focal neurological symptoms	2.31	0.49–10.94	0.290
Number of intrathecal injections	0.93	0.84–1.04	0.221
Driver mutation	0.46	0.13–1.68	0.244

* *p* < 0.05. Statistically significant *p* values are indicated by an asterisk. HR, hazard ratio; CI, confidence interval.

**Table 4:** Exploratory multivariable Cox proportional hazards analysis of overall survival from LM diagnosis in patients with lung adenocarcinoma harboring actionable driver mutations treated with targeted therapy (*n* = 46; 10 deaths). The four-variable model used for the nomogram, fitted from both time origins, is shown in Supplementary Table S4.

Variable	HR	95% CI	*p*-Value
Age	0.87	0.79–0.97	0.008 *
Epilepsy	0.44	0.02–10.49	0.613
Hydrocephalus	1.14	0.05–27.41	0.934
KPS	0.91	0.86–0.96	0.0003 *
Extracranial metastasis	8.92	1.06–75.03	0.044 *
Driver mutation	0.07	0.01–0.58	0.014 *

* *p* < 0.05. Statistically significant *p* values are indicated by an asterisk. HR, hazard ratio; CI, confidence interval.

## Data Availability

The data supporting the findings of this study are not publicly available due to patient privacy concerns but are available from the corresponding author (Hongqing Cai) upon reasonable request.

## Supplementary Materials

The following supporting information can be downloaded at: https://www.mdpi.com/article/10.3390/jcm15207808/s1, Figure S1. Overall survival from reservoir implantation and by tumour type; Figure S2. Dichotomized survival analyses in lung adenocarcinoma-associated LM measured from reservoir implantation; Figure S3. Exploratory prognostic model; Table S1. Clinical Information of 5 Patients with LM from Breast Cancer; Table S2. Cause of death in the 14 deceased patients; Table S3. Dichotomized Kaplan–Meier analyses in patients with lung adenocarcinoma-associated LM (*n* = 58), by time origin; Table S4. The four-variable Cox model used for the nomogram, fitted with overall survival measured from LM diagnosis and from reservoir implantation (lung adenocarcinoma with actionable driver mutations treated with targeted therapy, *n* = 46; 10 deaths).
