# Supplementary material for: Ommaya Reservoir-Based Cerebrospinal Fluid Chemotherapy for Leptomeningeal Metastasis in the Era of Targeted Therapy: Safety, Survival, and Exploratory Prognostic Factors from a Single-Center Cohort
Source: J Clin Med. 2026 Oct 10;15(20):7808. doi: 10.3390/jcm15207808 (PMC13705536; doi:10.3390/jcm15207808)
Supplement: Supplementary file 1 [file jcm-15-07808-s001.zip › Supplementary_Figures_S1-S3.pdf]

Supplementary Figures

Supplementary Figure S1. Overall survival from reservoir implantation and by tumour type

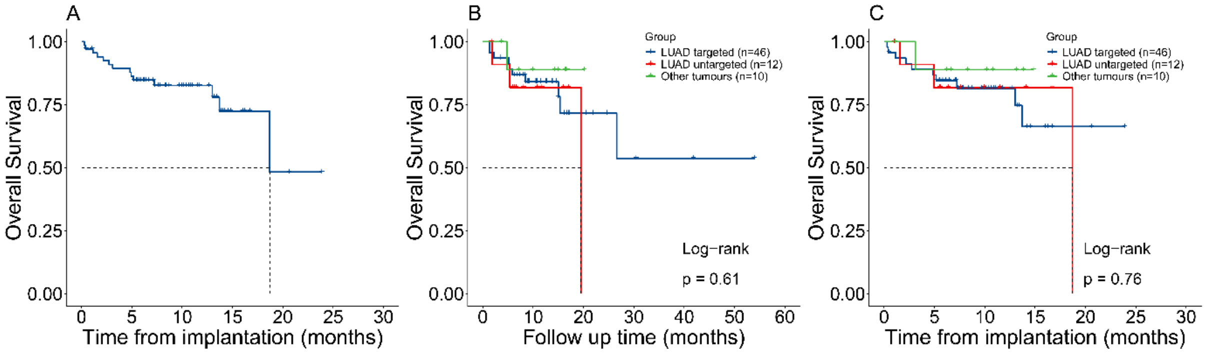

Supplementary Figure S1. Kaplan–Meier analyses of overall survival (OS). **A.** OS of the entire cohort (n = 68) measured from the date of Ommaya reservoir implantation; median OS 18.7 months (95% CI, 18.7 to not reached), with 6-, 12-, and 24-month OS of 84.7%, 82.6%, and 48.3%. **B.** OS from LM diagnosis and **C.** OS from reservoir implantation according to tumour type and treatment: lung adenocarcinoma with actionable driver mutations treated with targeted therapy (n = 46; 10 deaths), lung adenocarcinoma without targeted therapy (n = 12; 3 deaths), and other primary tumours (n = 10; 1 death). Log-rank p = 0.6 (B) and p = 0.8 (C); the number of events in the two smaller groups is too small for a meaningful comparison. Dashed lines indicate median survival. Abbreviations: LM, leptomeningeal metastasis; LUAD, lung adenocarcinoma.

Supplementary Figure S2. Dichotomized survival analyses in lung adenocarcinoma–associated LM measured from reservoir implantation

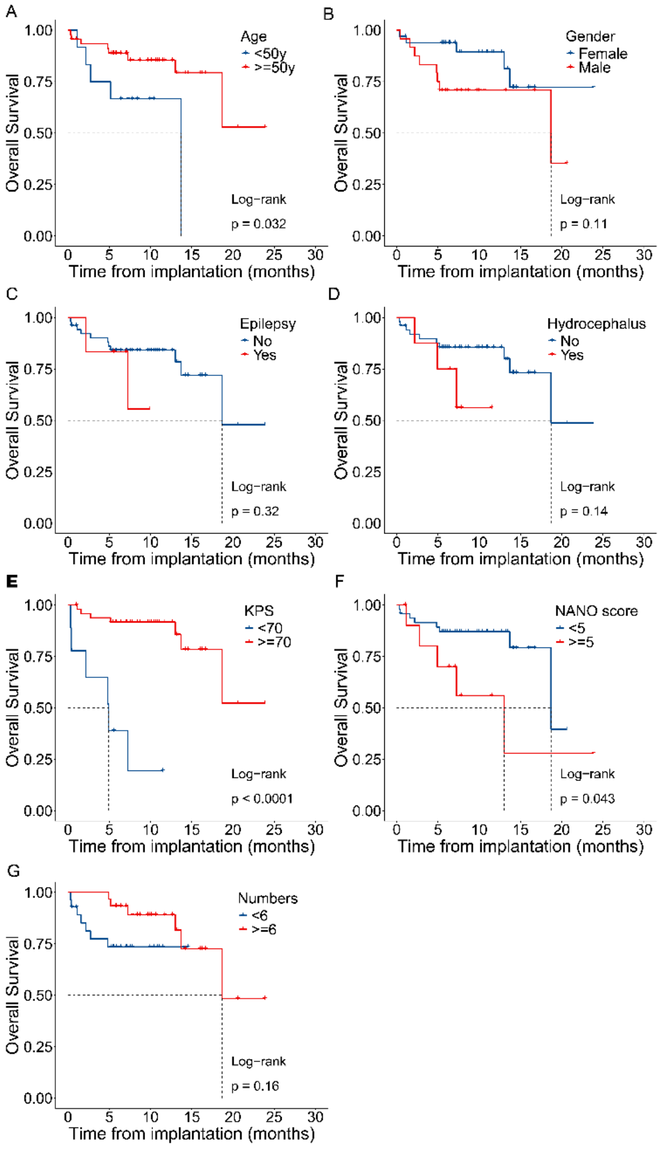

Supplementary Figure S2. Kaplan–Meier analyses of overall survival measured from the date of Ommaya reservoir implantation in patients with lung adenocarcinoma–associated LM (n = 58), corresponding to Figure 2B–H of the main text. **A.** Age (<50 vs ≥50 years; log-rank p = 0.032). **B.** Sex (p = 0.11). **C.** Epilepsy at LM diagnosis (p = 0.32). **D.** Hydrocephalus on neuroimaging at LM diagnosis (p = 0.14). **E.** Baseline KPS (<70 vs ≥70; p < 0.001). **F.** Baseline NANO score (≥5 vs <5; p = 0.043). **G.** Number of intrathecal injections (<6 vs ≥6; p = 0.155). P values were calculated using the log-rank test. Abbreviations: KPS, Karnofsky Performance Status; NANO, Neurologic Assessment in Neuro-Oncology; LM, leptomeningeal metastasis.

Supplementary Figure S3. Exploratory prognostic model

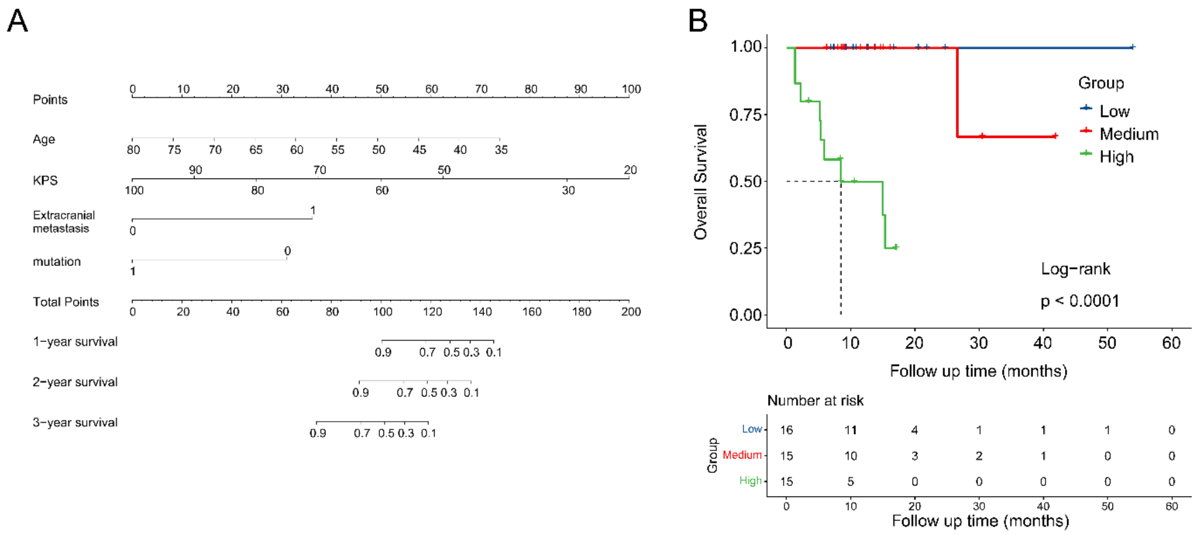

Supplementary Figure S3. Exploratory prognostic model in patients with lung adenocarcinoma–associated LM harboring actionable driver mutations and treated with targeted therapy (n = 46; 10 deaths); overall survival measured from LM diagnosis. **A.** Nomogram for 1-, 2-, and 3-year overall survival based on age, KPS, extracranial metastasis, and EGFR mutation subtype (classical EGFR mutation, exon 19 deletion or L858R, versus other); shown for illustration only, not validated. **B.** Kaplan–Meier curves stratified by model-derived risk tertiles of the linear predictor (low, medium, and high risk; 0, 1, and 9 deaths; log-rank $p < 0.001$). Because the risk groups were derived and evaluated in the same dataset, their separation does not indicate predictive validity. Abbreviations: KPS, Karnofsky Performance Status; LM, leptomeningeal metastasis.
