# Supplementary material for: Ommaya Reservoir-Based Cerebrospinal Fluid Chemotherapy for Leptomeningeal Metastasis in the Era of Targeted Therapy: Safety, Survival, and Exploratory Prognostic Factors from a Single-Center Cohort
Source: J Clin Med. 2026 Oct 10;15(20):7808. doi: 10.3390/jcm15207808 (PMC13705536; doi:10.3390/jcm15207808)
Supplement: Supplementary file 1 [file jcm-15-07808-s001.zip › Supplementary_Tables_S1-S4.pdf]

Supplementary Tables

Supplementary Table S1. Clinical Information of 5 Patients with LM from Breast Cancer

Patient	Age	KPS	Complications	Follow-up time (months)	Status
1	38	80	CSF infection	16.8	Alive
2	54	10	none	4.8	Dead
3	43	90	none	16.5	Alive
4	60	80	Probable methotrexate-related pseudotumoral demyelinating lesion	15.0	Alive
5	63	90	subdural effusion	20.1	Alive

Supplementary Table S2. Cause of death in the 14 deceased patients

**No.**	**Primary tumor**	**Driver alteration**	**KPS at implantation**	**OS from LM diagnosis (months)**	**OS from implantation (months)**	**Cause of death**
1	Lung adenocarcinoma	EGFR L858R	30	8.5	7.2	Systemic progression
2	Lung adenocarcinoma	ALK fusion	50	1.3	0.3	Systemic progression
3	Lung adenocarcinoma	KRAS	90	19.5	18.7	CNS progression
4	Lung adenocarcinoma	EGFR exon 19 deletion	80	26.6	13.0	CNS progression
5	Lung adenocarcinoma	EGFR exon 19 deletion	90	15.0	13.7	CNS progression
6	Lung adenocarcinoma	No actionable driver	20	5.4	4.9	CNS progression
7	Lung adenocarcinoma	EGFR exon 19 deletion + C797S	80	5.3	5.2	CNS progression
8	Lung	No actionable	90	1.9	1.6	CNS

	adenocarcinoma	driver				progression
9	Lung adenocarcinoma	EGFR, subtype unspecified	20	1.3	0.4	Systemic progression
10	Lung adenocarcinoma	EGFR exon 20 insertion	80	5.2	1.1	CNS progression
11	Lung adenocarcinoma	EGFR L858R	70	5.9	2.7	CNS progression
12	Lung adenocarcinoma	EGFR, subtype unspecified	60	2.2	2.2	Systemic progression
13	Lung adenocarcinoma	EGFR L858R	20	15.3	4.8	CNS progression
14	Breast cancer	ER+/PR+/HER2 2+	10	4.8	3.1	CNS progression

Cause of death was classified from medical records and telephone follow-up. No death was attributed to the reservoir or to intrathecal chemotherapy. Abbreviations: KPS, Karnofsky Performance Status; OS, overall survival; LM, leptomeningeal metastasis.

Supplementary Table S3. Dichotomized Kaplan–Meier analyses in patients with lung adenocarcinoma–associated LM (n = 58), by time origin

**Variable**	**Groups (n; deaths)**	**Median OS from diagnosis (months)**	**Log-rank p (from diagnosis)**	**Median OS from implantation (months)**	**Log-rank p (from implantation)**
Age	<50 (12; 5) vs ≥50 (46; 8)	15.0 vs NR	0.0095	13.7 vs NR	0.032
Sex	Male (24; 8) vs female (34; 5)	19.5 vs NR	0.073	18.7 vs NR	0.11
Epilepsy	Yes (6; 2) vs no (52; 11)	NR vs 26.6	0.22	NR vs 18.7	0.32
Hydrocephalus	Yes (8; 3) vs no (50; 10)	NR vs NR	0.080	NR vs 18.7	0.14
KPS	<70 (9; 6) vs ≥70 (49; 7)	8.5 vs NR	<0.001	4.9 vs NR	<0.001
NANO score	≥5 (11; 5) vs <5 (47; 8)	26.6 vs NR	0.054	13.0 vs 18.7	0.043
Intrathecal injections	≥6 (30; 6) vs <6 (28; 7)	NR vs NR	0.086	18.7 vs NR	0.155

NR, not reached. The number of intrathecal injections depends on survival time and is subject to immortal-time bias. Abbreviations: KPS, Karnofsky Performance Status; NANO, Neurologic Assessment in Neuro-Oncology.

Supplementary Table S4. The four-variable Cox model used for the nomogram, fitted with overall survival measured from LM diagnosis and from reservoir implantation (lung adenocarcinoma with actionable driver mutations treated with targeted therapy, n = 46; 10 deaths)

Variable	HR (95% CI), OS from diagnosis	p	HR (95% CI), OS from implantation	p
Age (per year)	0.89 (0.82–0.96)	0.004	0.91 (0.84–0.98)	0.012
KPS (per point)	0.91 (0.86–0.96)	<0.001	0.91 (0.87–0.96)	<0.001
Extracranial metastasis (yes vs no)	9.29 (0.96–89.9)	0.054	3.00 (0.38–23.6)	0.30
Classical EGFR mutation (19del/L858R vs other)	0.09 (0.01–0.64)	0.016	0.18 (0.03–0.98)	0.047
Harrell's C-index, apparent (SE)	0.945 (0.020)		0.943 (0.019)	
Harrell's C-index, bootstrap optimism-corrected	0.909		0.913	

Events per variable = 2.5. Optimism correction by bootstrap resampling (1000 resamples, rms::validate). The multivariable analysis from LM diagnosis that preceded model construction is shown in Table 4 of the main text. Abbreviations: HR, hazard ratio; CI, confidence interval; SE, standard error; OS, overall survival; KPS, Karnofsky Performance Status.
